# Impact of remote social interaction during the COVID-19 pandemic on the cognitive and psychological status of older adults with and without cognitive impairment: A randomized controlled study

**DOI:** 10.1371/journal.pone.0311792

**Published:** 2024-11-12

**Authors:** Ana L. Vives-Rodriguez, Anna Marin, Kylie A. Schiloski, Gabor P. Hajos, Adolfo Di Crosta, Irene Ceccato, Pasquale La Malva, Diana C. Anderson, Naheer Lahdo, Kaleigh Donnelly, Jiali Dong, Sabrina Kasha, Colleen Rooney, Judith Dayaw, Gabrielle Marton, Audrey Wack, Vanessa Hanger, Renée DeCaro, Alberto Di Domenico, Katherine W. Turk, Rocco Palumbo, Andrew E. Budson

**Affiliations:** 1 Center for Translational Cognitive Neuroscience, VA Boston Healthcare System, Boston, MA, United States of America; 2 Department of Neurology, Boston University School of Medicine, Boston, MA, United States of America; 3 Department of Psychology, G. d’Annunzio University of Chieti-Pescara, Chieti, Italy; 4 Alzheimer’s Disease Research Center, Department of Neurology, Boston University School of Medicine, Boston, MA, United States of America; Yonsei University - Wonju Campus, REPUBLIC OF KOREA

## Abstract

**Background:**

Social isolation and loneliness have both been associated with psychological health and cognitive decline in older adults. This study investigated the impact of social interaction through remote communication technologies during the COVID-19 pandemic on the cognitive and psychological status of older adults with and without cognitive impairment.

**Methods:**

Participants were recruited from Boston (USA) and Chieti (Italy). The study used a randomized single-blinded controlled crossover design with an intervention (remote social conversations with research staff over 20-minute video or telephone calls three times per week) and a passive control condition, each one of 4-weeks duration. The primary outcome was a composite cognitive score change from baseline to week 4. Secondary outcomes included scales for mood, anxiety, and loneliness.

**Results:**

Out of 196 participants recruited from April 2020 to April 2021, 17% dropped out. Based on the blind MoCA, 52% had cognitive impairment, and 25% were at risk of social isolation according to the Lubben social network scale. We observed that larger social networks were linked to better cognitive status and lower depression and anxiety levels, while loneliness was directly associated to depression severity. Older adults with cognitive impairment exhibited higher levels of depression and anxiety and were at greater risk for social isolation. In terms of the intervention, 91% preferred telephone over video calls. The intervention did not lead to improvements in cognitive or psychological scores.

**Conclusions:**

More work is needed to assess the utility of this intervention for the support of a heterogenous cross-cultural sample of older adults at-risk for social isolation, including individuals with cognitive impairment. Future research should explore longer intervention periods, categorize participants by call type, and target those meeting social isolation criteria.

**Trial registration:**

ClinicalTrials.gov NCT04480112.

## Introduction

In recent decades, there has been a great interest in understanding the role of social isolation and loneliness in the health and well-being of older adults. Social isolation is an objective measurement of the individual’s social connections, whereas loneliness is the perceived discrepancy between the individual’s desired and actual social network [1, 2]. The prevalence of social isolation is between 6–20% in older adults [1]. In contrast, the prevalence of loneliness is between 20–40% of older adults 50 to 80 years old [3] and up to 50% in older adults above 80 years old [4].

Meaningful social connections have been associated with better health outcomes, improved quality of life, and a 50% increase in survival [5]. Both social isolation and loneliness have been studied as risk factors for cognitive decline later in life [1]. Studies have associated loneliness and social isolation with (1) lower cognitive scores and more rapid cognitive decline [2, 6] (2) with a higher risk for depression [7], and (3) with a diminished quality of life in the older population [8], especially in individuals with cognitive impairment [9].

Remote communication devices—such as mobile phones, video conferencing, and digital tablets—have been implemented in prior studies as a surrogate for in-person social interaction to treat social isolation and loneliness in older adults [10]. In general, these studies implemented the intervention while the participants were in their usual living environments, without the restrictions of in-person social interactions such as those imposed by the pandemic [10].

Results have been limited on the effect of remote communication technologies on the cognitive and psychological status of older adults with cognitive impairment [11–13]. Although little attention has been given to social isolation in the aging population with cognitive impairment, recent work is showing that cognitively impaired older adults are more vulnerable to social isolation and loneliness, and have diminished compensatory mechanisms to deal with the cognitive and psychological detrimental effects resulting from them [11]. As a results, the COVID-19 policies to ensure public safety created a unique model to study the effect of social isolation on cognition and mood in older adults.

The primary objectives of our study were to: (1) assess the relationship between social isolation and loneliness with cognition and mood (depression and anxiety) in our heterogenous sample of older adults with and without cognitive impairment during the COVID-19 pandemic; (2) investigate the feasibility and effectiveness of frequent social interaction through remote communication technologies for four weeks on the cognitive and psychological status of older adults with and without cognitive impairment.

We hypothesized that the degree of social isolation and loneliness would be directly associated with the degree of cognitive impairment, depression, and anxiety during the social isolation period of the pandemic. We also hypothesized that our frequent social interactions schedule would be feasible in the older population and that, given the detrimental effects of social isolation on cognitive and psychological health, engagement in frequent social interactions would have a beneficial effect on older adults’ degree of cognitive impairment, depression, and anxiety.

## Methods

### 1. Study population

Participants were recruited from two research sites: Boston, Massachusetts, USA and Chieti, Italy. In Boston, participants were recruited from a tertiary memory disorders clinic at the Veterans Affairs (VA) Boston Healthcare System and from volunteers who responded to advertisements at Boston University Alzheimer’s Disease Research Center. In Chieti, participants responded to advertisements at G. d’Annunzio University of Chieti-Pescara. The study was approved by Boston University Institutional Review Board (IRB; study protocol H-26786), VA Boston Healthcare System IRB (study protocol 3313), and the Ethical Committee of the Department of Psychological, Health and Territorial Sciences, University “G. d’Annunzio” (study protocol 20004). Participants provided either verbal or written informed consent depending on the time they started their participation in the study. Time of consent and name of research staff member witnessing consent was recorded as proof of verbal consent.

Participants were enrolled if they were 50 years old or older and had access to either a computer, smart device, or telephone. Internet connectivity was not required to participate. Based on their initial blind Montreal Cognitive Assessment (MoCA-B) [14], we included older adults without cognitive impairment (score 18 or above) and older adults with cognitive impairment (score 17 or below) ranging from mild cognitive impairment to mild dementia.

**Fig 1:**
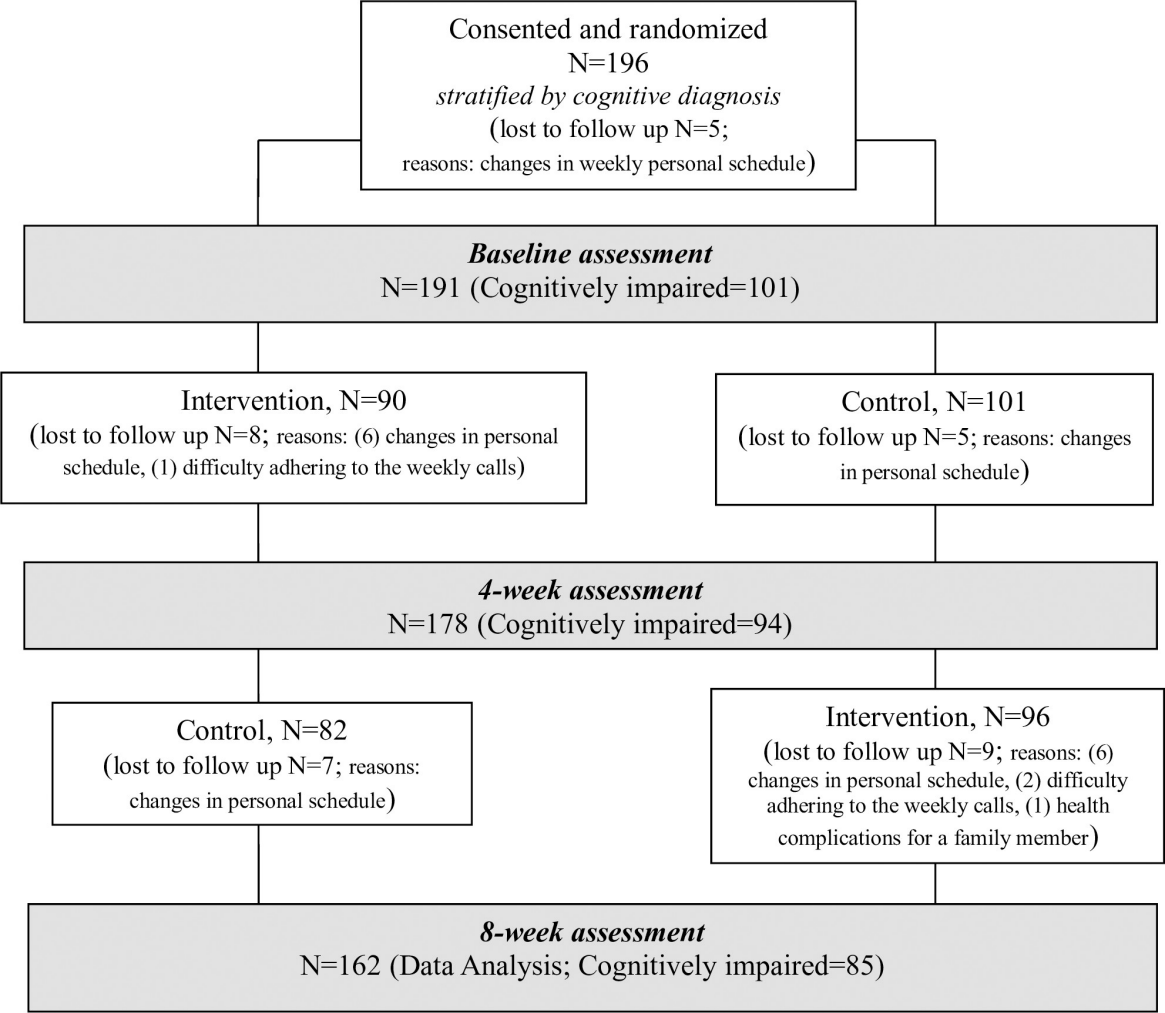
Consort flowchart.

From May 2020 to April 2021, we recruited 196 participants. 17% of participants withdrew from the study (34/196) over the two months (see Fig 1).

Data collected in Boston (USA) and Chieti (Italy) was compared to account for COVID-related timeline and policy variations between the two countries [15–18]. Italy was one of the first countries impacted by the pandemic and it implemented strict lockdown measures nationally, whereas the US adopted more gradual state-specific policies [19–21]. In addition, an interaction has been found between cultural factors and containment policies between the two countries, which emphasizes the importance of comparing the Italian and American cohorts [22].

Enrollment in Boston (USA) began on 06/02/2020 and ended on 4/14/2021. Enrollment in Chieti (Italy) began on 6/15/2020 and ended on 12/01/2020.

### 2. Study design

This was a randomized single-blinded controlled intervention study using an AB/BA crossover design (2-sequence, 2-period, 2-treatment) (see Fig 1).

Participants were assigned to start with either the intervention or the control condition. Randomization was stratified by cognitive diagnosis (i.e., cognitive impaired and cognitive unimpaired) determined by their initial MoCA-B score. An independent researcher, not otherwise involved in the study, generated the randomization sequence using permuted blocks technique. All other assessors remained blinded to the allocation of participants.

### 3. Intervention and control conditions

In the intervention phase, participants engaged in 20-minute remote social conversations with research staff members three times per week via video or telephone based on participants’ technology availability and preference. Research staff followed a scripted guide (see S1 File) designed to promote pleasant conversation covering topics like participants’ remote history, current events, hobbies, and more, including spontaneous topics like future life plans and COVID-19. To ensure participant engagement and a positive experience, the content of each call varied based on individual preferences and daily conversational interests. This approach aimed to mitigate potential burden, particularly in light of the unprecedented social stressors introduced by the COVID-19 pandemic. The format and application method of the intervention were motivated by previous research evaluating the effectiveness of various methods using information communication devices to alleviate social isolation in older adults [10], as well as by a specific study examining the impact of a telephone-based befriending program in supporting socially isolated older adults [23]. Out of a sample of available qualitative data (31% of all calls), approximately 17% of calls included the topic of the pandemic.

**Fig 2:**
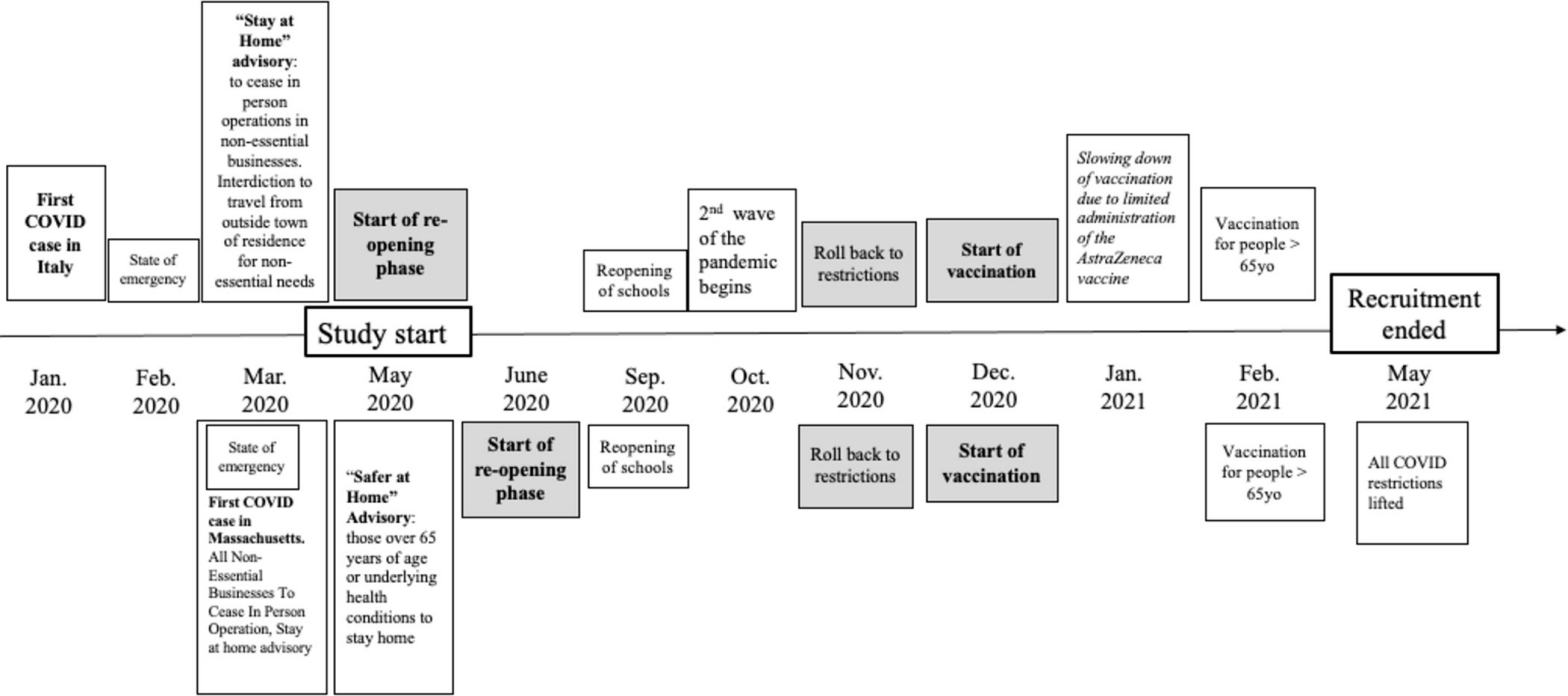
Timeline of the COVID-19 pandemic in relation to the study’s recruitment period.

During the control phase, participants had no contact with the research team. They were instructed to return to their regular routines, which, at the time, meant adhering to stay-at-home and social-distancing advisories from local healthcare authorities. Health policies varied between Italy and the United States during the study period (see Fig 2).

We excluded individuals who couldn’t understand the informed consent due to cognitive or hearing impairment or who had a history of clinically significant depression, alcohol or drug use, cerebrovascular disease, traumatic brain injury, or any non-neurological condition significantly impairing cognition (e.g., organ failure).

### 4. Cognitive and psychological outcome measures

#### 4.1 Primary outcome

The primary outcome was the change in the cognitive composite score from baseline to week 4, comparing participants who engaged in the social interventions and those who did not. The composite score was based on 5 subtests from the Repeatable Battery for the Assessment of Neuropsychological Status (RBANS) [24] administered by telephone, plus oral trail making (OTMT) parts A and B [25].The RBANS subtests were: list learning, delayed recall, recognition, category fluency, and digit span (total maximum score = 126 points); higher scores indicate, better performance. The RBANS has been previously validated for remote administration in adults over age 55 with and without cognitive impairment with correlations compared to in-person administration that ranged from 0.75 to 0.90 for the subtests used for this study [26].

OTMT parts A and B were scored both for completion time and the number of errors, where shorter times and fewer errors reflected better performance. OTMT-B is a validated alternative to written TMT-B, correlating highly and activating dorsolateral and medial prefrontal cortices in functional MRI [25, 27, 28].

To calculate the cognitive composite score, we transformed the seven cognitive tasks into z-scores. For OTMT parts A and B, we reversed the z-scores by multiplying by -1. We then summed the standardized scores for each test and divided by seven (total number of tests) to obtain an unweighted average.

#### 4.2 Secondary outcomes

Secondary outcomes included: changes from baseline to week 4 in psychological symptoms measured by the Geriatric depression scale–short form (GDS) [29, 30], Geriatric Anxiety Inventory (GAI) [31, 32], and the UCLA loneliness scale [33, 34].The GDS-short form maximum score is 15, with scores above 5 suggesting depression. The GAI maximum score is 20, with higher scores indicating more anxiety symptoms. The UCLA Loneliness scale has a range of 20 to 80, where higher scores indicate greater loneliness. The Lubben social network scale (LSNS) [35, 36], was used for assessing baseline social isolation and evaluating social networks during the study, not as an outcome measure. LSNS scores range from 0 to 30, with scores of 12 or lower indicating a risk for social isolation.

### 5. Data analysis

A power analysis was conducted using G-Power [37]. Alpha was set to 0.05, and Beta to 0.80. Studies with similar methodologies found small to medium effect sizes [38] when reporting differences between individuals completing the intervention and the control conditions. We performed our power analysis for an independent t-test. It was determined that for a medium effect size (0.5), a sample of 64 participants would be needed in each group, and for a small effect size (0.35), a sample size of 130 in each group would be needed. Some prior studies testing the effect of social interactions in older adults have used sample sizes of ranging between 100 and 150, participants per group, which is in keeping with our estimated necessary sample size [39, 40].

Data analyses were performed using Stata 16 (Version IC 16.1).

For aim 1, we employed T-tests, Mann-Whitney U tests and Chi-square tests to compare participants with and without cognitive impairment (α = 0.05) and between the US and Italian samples. We used Pearson correlations and linear regressions to assess the relationships between social isolation (LSNS), loneliness (UCLA loneliness scale), and baseline cognitive (RBANS, OTMT) and psychological measures (GDS, GAI).

To address aim 2, adherence was evaluated by tracking participant enrollment during the active and passive control periods. T-tests were used to analyze all change-from-baseline scores for primary and secondary outcome measures.

## Results

### 1.1 Characteristics of study participants

Table 1 displays the demographics of our sample. The sample had a mean age of 74.26 (±8.02; range: 53–95) and was more than half male (58.02%, 94/162) with an average of 11.75 years of education (±4.62; range: 3–24). Most participants were retired (retired: 79.01%, 128/162; employed: 11.72%, 19/162; unemployed: 9.26%, 15/164), and married or partnered (married: 58.64%, 95/162; widowed: 24.69%, 40/162; divorced or separated: 10.49%, 17/162; single: 6.17%, 10/162). About 29.01% (47/162) lived alone, and 84.57% (137/162) were independent in all their instrumental activities of daily living. The mean social isolation score was 15.98 (±6.12; range: 0–30) with 25.31% (41/162) at risk for social isolation based on the LSNS. The mean loneliness score was 36.44 (±7.59; range:16–54). GDS and GAI scores averaged 4.15 (±2.71; range: 0–13) and 4.77 (±5.16; range: 0–19), with clinically significant depression in 33.33% (54/162) and anxiety in 25.92% (42/162) of participants, respectively. Median MoCA-B score was 16.85 (±3.49; range: 7–22) with 52.47% (85/162) scoring in the impaired range (17 or below). Ninety-one percent (148/162) preferred telephone calls for the intervention. Most participants completed the study before a COVID-19 vaccine was available (76.54%, 124/162). No differences were found between participants living alone versus with others, or between married and unmarried individuals.

**Table 1:** Baseline data.

Characteristics	Total sample	Group 1	Group 2
	N = 162	N = 75	N = 87
Age in years	74.26 ±8.02	74.7 ±8.09	73.9 ±7.98
	(53–95)	(53–94)	(62–95)
Male gender	94 (58.02)	24 (32.00)	33 (37.93)
Years of education	11.75±4.62	12.12±4.80	11.42±4.45
	(3–24)	(4–24)	(3–20)
Occupation			
Retired	128 (79.01)	55 (73.33)	67 (77.01)
Employed	19 (11.72)	11 (14.67)	8 (9.19)
Unemployed	15 (9.26)	9 (12.00)	6 (6.90)
Marital Status			
Married or partnered	95 (58.64)	40 (53.33)	55 (63.22)
Widowed	40 (24.69)	19 (25.33)	18 (20.67)
Divorced or separated	17 (10.49)	11 (14.67)	6 (6.90)
Single	10 (6.17)	5 (6.67)	5 (5.75)
Living situation			
Alone	47 (29.01)	25 (33.33)	22 (25.29)
With someone	115 (70.99)	50 (66.67)	65 (74.71)
Blind MoCA	16.85 ±3.49 (7–22)	16.80 (3.44)	16.88 (3.55)
Impaired score (≤17)	85 (52.47)	40 (53.33)	45 (51.72)
Functionality			
Dependent in any iADL	26 (16.05)	7 (9.33)	19 (25.33)
Dependent in any bADL	2 (2.60)	1 (1.33)	1 (1.33)
Independent ambulation	137(84.57)	64 (85.3)	73 (97.33)
Lubben Social Network Scale	15.98 ± 6.12 (0–30)	16.31 ± 5.84 (7–30)	15.69 ± 6.37 (0–29)
% at risk for social isolation	41 (25.31)	17 (22.67)	24 (27.59)
UCLA Loneliness Scale	36.44 ±7.59	35.65±7.63	37.13±7.54
	(16–54)	(16–49)	(20–54)
Geriatric Depression Scale	4.15 ±2.71 (0–13)	3.83 ±2.47 (1–12)	4.42 ±2.89 (0–13)
% with clinical depression	54 (33.33)	22 (29.33)	32 (36.78)
Geriatric Anxiety Inventory	4.77 ±5.16 (0–19)	5.09 ±5.34 (0–19)	4.48 ±5.02 (0–18)
% with clinically significant anxiety	42 (25.92)	23 (30.67)	19 (21.84)
% who preferred telephone calls over video	148 (91.36)	67 (89.33)	81 (93.10)
% who completed the study before the COVID vaccine	124 (76.54)	59 (78.67)	65 (74.71)

Values represent number (percentage) and means ± standard deviation (range);MoCA = Montreal Cognitive assessment, UCLA = University of California Los Angeles, iADL = instrumental activities of daily living, bADL = basic activities of daily living

As shown in Table 2, participants with cognitive impairment had a mean MoCA-B score of 14, fewer years of education, higher depression and anxiety scores, and a smaller social network size compared to those without cognitive impairment.

**Table 2:** Baseline data compared between older adults with and without cognitive impairment.

Characteristics	Cognitively unimpaired	Cognitively impaired	Test statistic	*p*
	n = 77	n = 85		
Age in years	74.10 ±8.96	74.4 ±7.10	*t* = -0.231	0.82
Male gender	49 (63.64)	45 (52.94)	χ^2^ = 1.897	0.20
Years of education	13.69 ±4.14	9.99 ±4.33	*t* = 5.544	<0.001
Occupation				
Retired	52 (72.22)	70 (83.33)	χ^2^ = 3.768	0.21
Employed	12 (16.67)	7 (8.33)		
Unemployed	8 (11.11)	7 (8.33)		
Marital Status				
Married or partnered	41 (53.25)	54 (63.53)	χ^2^ = 1.947	0.30
Widowed	21 (27.28)	19 (22.35)		
Divorced or separated	9 (11.69)	8 (9.41)		
Single	6 (7.79)	4 (4.71)		
Living situation				
Alone	25 (34.72)	22 (26.19)	χ^2^ = 1.341	0.25
With someone	47 (65.28)	62 (73.81)		
Blind MoCA	20.02 ±1.31	14 ±2.09	*t* = 22.197	<0.001
Impaired score (≤17)				
Functionality				
Dependent in any iADL	15(19.48)	11(12.94)	χ^2^ = 1.282	0.26
Dependent in any bADL	2 (2.60)	0 (0.00)	χ^2^ = 2.235	0.22
Independent ambulation	61 (80.26)	76 (89.41)	χ^2^ = 2.648	0.10
Lubben Social Network Scale	18.03 ±6.11	14.11 ±5.53	*U = 2032*.*0*	<0.001
% at risk for social isolation	10 (12.99)	31 (36.47)		
UCLA Loneliness Scale	34.88 ±7.33	37.80 ±7.60	*U* = 3882.5	0.011
Geriatric Depression Scale	3.19 ±1.97	5.01 ±3.00	*U = 4514*.*0*	<0.001
% with clinical depression	18 (23.38)	36 (42.35)		
Geriatric Anxiety Inventory	2.73±3.66	6.61±5.63	*U = 4720*.*0*	<0.001
% with clinically significant anxiety	9 (11.69)	33 (38.82)		
% who preferred telephone calls over video	65 (84.42)	83 (97.65)	χ^2^ = 8.959	.003
% who completed the study before the COVID vaccine	53 (68.83)	71 (83.53)	χ^2^ = 4.861	.027

Values represent number (percentage) and means ± standard deviation (range); Independent t-tests were run for continuous variables (t), Mann-Whitney U tests were run for ordinal variables (*U*), and Chi-square tests were run for proportional variables; MoCA = Montreal Cognitive assessment, UCLA = University of California Los Angeles, iADL = instrumental activities of daily living, bADL = basic activities of daily living

Compared to the Boston cohort, the Italian cohort had lower educational attainment, lower scores on the MoCA-B, higher depression and anxiety symptoms, and a smaller social network size (see Table 3 for details).

**Table 3:** Descriptive data comparing participants from Italy and United States.

Characteristics	Italy	United States	Test statistic	*p*
	(N = 112)	(N = 50)		
Age in years	73.08 (±7.17)	76.90 (±9.19)	*t* = -2.863	0.005
Female gender	71 (63.39)	23 (46.00)	χ^2^ = 4.293	0.038
Years of education	9.81 (±3.83)	16.08 (±3.02)	*t* = -11.192	<0.001
Occupation				
Retired	86 (76.78)	41 (82.00)	χ^2^ = 6.794	0.079
Employed	12 (10.71)	7 (14.00)		
Unemployed	14 (12.50)	1 (2.00)		
Marital Status				
Married or partnered	67 (59.82)	28 (56.00)	χ^2^ = 0.648	0.885
Widowed	28 (25.00)	12 (24.00)		
Divorced or separated	11 (9.82)	6 (12.00)		
Single	6 (5.36)	4 (8.00)		
Living situation				
Alone	32 (28.57)	15 (3.00)	χ^2^ = 0.457	0.499
With someone	80 (71.43)	29 (58.00)		
Blind MoCA	15.94 (±3.21)	18.92 (±3.24)	*t* = -5.405	<0.001
Functionality				
Dependent in any iADL	6(5.36)	20 (40.00)	χ^2^ = 30.791	< .001
Dependent in any bADL	1 (0.89)	1 (2.00)	χ^2^ = 0.348	0.556
Independent ambulation	105 (93.75)	32 (64.00)	χ^2^ = 21.741	<0.001
Lubben Social Network Scale	14.73 (±5.38)	18.76 (±6.77)	*U = 3820*.*5*	<0.001
% at risk for social isolation	31 (27.68)	10 (20.00)		
UCLA Loneliness Scale	36.76 (±7.06)	35.68 (±8.78)	*U* = 2402.0	0.385
Geriatric Depression Scale	4.67 (±2.89)	2.98(±1.79)	*U = 1812*.*5*	<0.001
% with clinical depression	43 (38.39)	11 (22.00)		
Geriatric Anxiety Inventory	6.16(±5.38)	1.64 (±2.74)	*U = 1130*.*5*	<0.001
% with clinically significant anxiety	38 (33.93)	4 (8.00)		

Values represent number (percentage) and means ± standard deviation (range); Independent t-tests were run for continuous variables (t), Mann-Whitney U tests were run for ordinal variables (*U*), and Chi-square tests were run for proportional variables; MoCA = Montreal Cognitive assessment, UCLA = University of California Los Angeles, iADL = instrumental activities of daily living, bADL = basic activities of daily living

### 1.2 Measures of social isolation at baseline and their relationship to initial cognitive and psychological variables

To assess social isolation, we examined both social network size using the Lubben social network scale and the perceived degree of loneliness using the UCLA loneliness scale. We investigated their associations with the following variables: score on the MoCA Blind, levels of depression measured by the GDS, and levels of anxiety measured by the GAI.

#### 1.2.a Degree of social isolation

As shown in Table 4, living alone was not associated with a smaller social network measured by the LSNS (OR = 1.01; *p* = 0.768; CI = 0.95, 1.07). Lower social network scores were associated with higher levels of depression and anxiety at baseline after controlling for loneliness (*r =* -0.12; *p*<0.001; CI = -0.19, -0.06 and *r =*: -0.27; *p*<0.001; CI = -0.39, -0.15, respectively), and they were associated with lower MoCA-B scores after controlling for loneliness, age, and years of education (*r =* 0.15; *p*<0.001; CI = 0.06, 0.23).

**Table 4:** Associations between measures of social isolation and baseline cognitive and psychological variables.

Variable 1	Variable 2	Covariates	Effect size	*p*	95% CI
**Social Network Score (LSNS)**	Living alone		OR = 1.01	0.768	0.95, 1.07
	Depression (GDS)*	*Loneliness*	*r* = -0.12	<0.001	-0.19, -0.06
	Anxiety (GAI)*	*Loneliness*	*r* = -0.27	<0.001	-0.39, -0.15
	MoCA-B*	*Loneliness Age YoE*	*r* = 0.15	<0.001	0.06, 0.23
**Feelings of Loneliness (UCLA Loneliness Scale)**	Social network Score (LSNS)		*r* = 0.14	0.07	-0.29, 0.02
	Living alone		OR = 0.99	0.743	0.95, 1.04
	Depression (GDS)*		*r* = -0.12	<0.001	-0.19, -0.06
	Anxiety (GAI)		*r* = 0.07	0.202	-0.04, 0.17
	MoCA-B		*r* = -0.05	0.101	-0.12, 0.01

#### 1.2.b Feelings of loneliness

As shown in Table 4, feelings of loneliness measured by the UCLA loneliness scale were not associated with social network size measured by the LSNS (*r* = 0.14; *p* = 0.07). Living alone was not associated with a higher level of loneliness (OR = 0.99; *p* = 0.743; CI = 0.95, 1.04). A higher level of loneliness was associated with depression (*r* = -0.12; *p*<0.001; CI = -0.19, -0.06) but not with anxiety levels (*r =* 0.07; *p* = 0.202; CI = -0.04, 0.17). No significant association was observed between feelings of loneliness and baseline MoCA-B scores (*r =* -0.05; *p* = 0.101; CI = -0.12, 0.01).

### 2.1 Adherence

As depicted in Fig 1, the intervention group exhibited dropout rates of 2.17% at baseline, 8.89% during month 1, and 8.54% during month 2. In contrast, the control group had dropout rates of 2.88% at baseline, 4.95% during month 1, and 9.37% during month 2.

### 2.2 Primary and secondary outcomes

Tables 5 and 6 display changes from baseline to 4 weeks for the composite cognitive score and the psychosocial outcome measures (UCLA Loneliness scale, GDS, GAI) when comparing the intervention and control conditions. Paired t-test results are reported to illustrate differences in score changes between the intervention group and baseline, as well as between the control group and baseline.

**Table 5:** Pre and post-intervention scores for the primary and secondary outcomes.

Total sample	N = 162	Mean	SD	Min	Max	Change from baseline	*t*	*df*	*p*
**cognitive z score**	*baseline*	0.000	0.689	-1.62	1.45		-.485	161	.629
	*post-intervention*	-0.001	0.727	-2.56	1.43	-0.001			
	*post-control*	0.010	0.730	-2.47	1.41	0.010			
**UCLA**	*baseline*	36.44	7.59	16	54		-1.334	161	.184
	*post-intervention*	34.57	8.13	20	60	-1.87			
	*post-control*	35.31	7.92	20	53	-1.13			
**GDS**	*baseline*	4.15	2.71	0	13		2.256	161	.025
	*post-intervention*	3.95	2.56	1	13	-0.2			
	*post-control*	3.68	2.25	0	12	-0.47			
**GAI**	*baseline*	4.76	5.16	0	19		2.276	161	.024
	*post-intervention*	4.21	4.69	0	19	-0.55			
	*post-control*	3.68	4.24	0	19	-1.08			

**Table 6:** Pre and post intervention for the primary and secondary outcomes in the sub-groups of participants with and without cognitive impairment.

Cognitively impaired	N = 85	Mean	SD	Min	Max	Change from baseline	*t*	*df*	*p*
**cognitive z score**	*baseline*	-0.431	0.621	-2.29	0.66		-.153	84	.879
	*post-intervention*	-0.291	0.796	-3.16	1.15	0.14			
	*post-control*	-0.312	0.798	-4.32	1.01	0.12			
**UCLA**	*baseline*	37.8	7.60	16	52		-1.577	84	.119
	*post-intervention*	36.26	8.35	20	60	-1.54			
	*post-control*	37.4	7.29	23	53	-0.4			
**GDS**	*baseline*	5.01	3.00	1	13		2.454	84	.016
	*post-intervention*	4.58	2.83	1	13	-0.43			
	*post-control*	4.13	2.55	0	12	-0.88			
**GAI**	*baseline*	6.61	5.63	0	19		2.833	84	.006
	*post-intervention*	6.03	5.15	0	19	-0.58			
	*post-control*	5.05	4.49	0	19	-1.56			
**Cognitively unimpaired**	**N = 77**	**Mean**	**SD**	**Min**	**Max**	**Change from baseline**	*t*	***df***	***p***
**cognitive z score**	*baseline*	0.242	0.249	0.07	0.42		-.562	76	.576
	*post-intervention*	0.215	0.334	-0.02	0.45	-0.027			
	*post-control*	0.245	0.504	-0.11	0.6	0.003			
**UCLA**	*baseline*	34.88	7.33	20	54		-.350	76	.728
	*post-intervention*	33.04	7.05	20	47	-1.84			
	*post-control*	33.3	7.55	20	48	-1.58			
**GDS**	*baseline*	3.19	1.97	0	10		.649	76	.518
	*post-intervention*	3.27	2.07	1	12	0.08			
	*post-control*	3.20	1.77	1	10	0.01			
**GAI**	*baseline*	2.73	3.66	0	15		.200	76	.842
	*post-intervention*	2.18	3.15	0	15	-0.55			
	*post-control*	2.14	3.42	0	18	-0.59			

## Discussion

Our hypothesis was that social isolation and loneliness would be associated with the cognitive and psychological status of older adults during the social isolation period of the pandemic. We also hypothesized that engagement in frequent social interactions through remote communication technologies would be feasible for an heterogenous sample of older adults with and without cognitive impairment and that it would benefit performance on cognitive domains—such as attention, executive function, and processing speed—as well as with feelings of loneliness, and mood.

**Fig 3:**
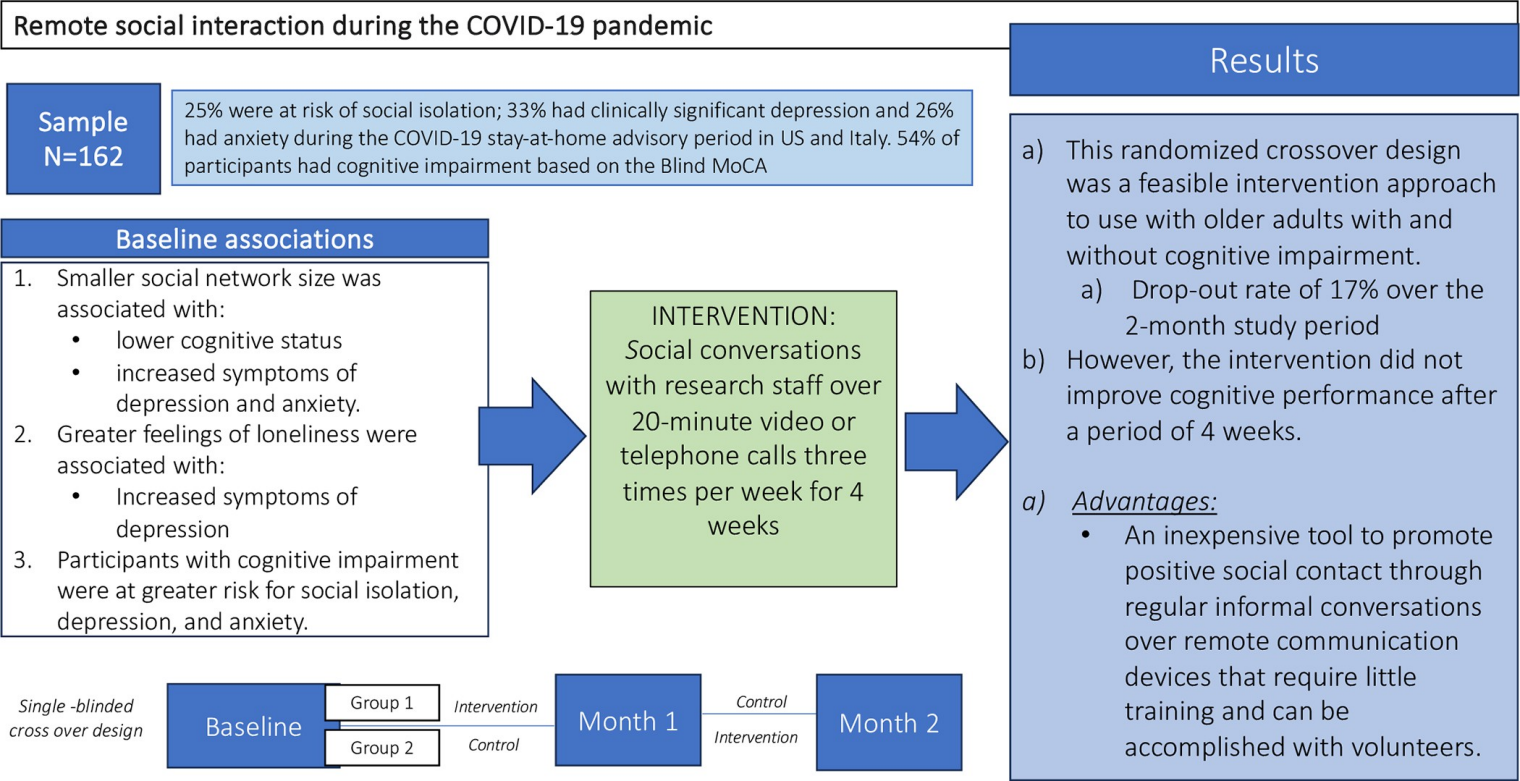
Study results.

We found that a smaller social network size was associated with lower cognitive status (measured by the blind MoCA) and more depression and anxiety symptoms (measured by the GDS and GAI) in our sample. We also found that higher ratings of loneliness were associated with more depression symptoms. Our results are both consistent with and add to the prior literature. Multiple studies have found that individuals who report fewer social interactions have higher symptoms of depression [41]. Additionally, older adults with cognitive impairment are at increased risk for social isolation, depression, and other neuropsychiatric symptoms [42, 43]. In our study, participants with cognitive impairment showed the highest risk for depression, anxiety, and social isolation, making this subgroup of particular interest for interventions with high accessibility like the one proposed (see Fig 3 for a summary of the results).

As a consequence of the pandemic, there has been an expansion of the use of remote communication technologies for all aspects of health care. However, there are often factors impacting widespread access, such as limited insurance coverage, high out-of-pocket costs for users, or patients’ hesitancy to engage in formal remote treatment. This type of intervention, consisting of informal conversations that promote positive social contact and build rapport over time, is inexpensive since it requires little training and could be implemented with volunteers. However, in this randomized, single-blinded controlled interventional study, regular social interaction through remote communication devices did not show an improvement in cognitive performance or mood after a period of 4 weeks.

Prior literature found that remote interventions for the treatment of social isolation in older adults improved loneliness, depression, and anxiety [44–46]. However, most of the available literature focuses on clinician-led interventions such as cognitive behavioral therapy or occupational therapy [39, 44–47].

### Limitations

There are several possible reasons why our intervention did not show an impact on our primary outcome measures of cognition, and had unexpected effects on feelings of depression and anxiety. First, in retrospect, it is likely that the duration (four weeks), frequency (three times a week), and dose (20 minutes each call) of our intervention were too limited to see a significant effect on cognition. However, given that 17% of participants were lost to follow-up, future studies should first examine how a more intensive frequency and dose of intervention could improve the benefit of the intervention, without increasing the length of the study to avoid a greater dropout rate.

Moreover, the social intervention involved spontaneous conversations guided by a script, which was utilized differently by the research staff based on the participants’ preferred topics of discussion. This intervention format introduces additional variability in the intervention’s impact on cognition. Future research should explore the efficacy of a structured interview approach, consistently applied to all participants over the four weeks, to ensure uniform coverage of conversation topics and questions. Future studies should also consider the addition of a washout period in between the social intervention and the control phase, to ensure as much as possible that the social intervention does not interact with the control phase. Finally, group-based interventions among older adults rather than individual participants socializing with study staff, could be investigated as they may foster more community building among older adults and further impact social isolation and loneliness measures.

Furthermore, the rapid changes in the social distancing recommendations during the study’s 12 month-period might have added heterogeneity to our sample and could have limited the effect of our intervention. Nevertheless, 85% of the participants concluded the study before vaccination was available.

During the control phase, participants were instructed to resume their regular routines. However, as we did not maintain contact with them during this phase, changes in social life may have occurred due to alterations in social distancing recommendations or personal circumstances that were not accounted for in the study. Future research should consider assessing these changes using a tailored daily or weekly questionnaire focused on daily social habits.

Our sample included participants from both Italy and the United States, introducing cultural and pandemic timing variations. In addition, our randomization was not stratified by country, potentially biasing our overall sample. Considering some of the differences we observed in the two populations, future studies should stratify randomization based on both cognitive status and country. Despite this complexity, our cross-cultural dataset offers valuable insights into social isolation and loneliness among older adults in both countries heavily affected by COVID-19.

Most participants chose to have telephone interactions rather than video calls. It is possible that voice calls did not provide sufficient social contact to impact loneliness and cognition. However, we did not exclude participants with only telephone availability since these were potentially the participants with the highest level of social isolation and we allowed participants to choose the preferred modality for the intervention to help promote a positive experience for them. Most participants selected telephone as opposed to video calls because they did not own a smartphone, tablet, or computer (or if they did, they did not feel comfortable using those devices frequently). Future studies should consider providing smartphones or tablets to participants, and should also implement training sessions on how to use the preferred communication device prior to the start of the intervention period [48, 49].

Although many participants had clinically relevant impairments (e.g., 52% impaired MoCA, 33% depression, 26% anxiety), these levels might not have been severe enough to detect intervention-related changes on cognition. Future research should include participants with lower baseline cognitive scores to assess intervention effectiveness across different stages of cognitive impairment. Additionally, longer mood questionnaires (e.g., GDS-long form) should be considered to better evaluate changes in psychological scores. Only 25% of participants were at risk for social isolation, potentially limiting our intervention’s impact due to the high level of social interaction outside of the study.

## Conclusion

The current study provides useful information for the development of a non-clinician-based social engagement intervention to support older adults at-risk for social isolation. Particularly this type of intervention appears feasible also for older individuals with cognitive impairment. This intervention may represent an inexpensive and convenient type of social engagement tool to support older adults across various levels of cognitive function. Nevertheless, limitations in our study design and the heterogeneity of our sample compounded by changes in social distancing recommendations throughout the study period and across the two countries, did not provide evidence to prove the intervention’s effectiveness on cognition and mood in older adults with and without cognitive impairment. Further research is needed to enhance participant adherence to the intervention schedule, examine its effects on cognitive and psychological health, and investigate its long-term impact on older adults vulnerable to social isolation.

## Supporting information

S1 File Intervention script. (PDF)

S2 File CONSORT checklist. (PDF)

S3 File Study protocol. (PDF)
