# Supplementary material for: Impact of remote social interaction during the COVID-19 pandemic on the cognitive and psychological status of older adults with and without cognitive impairment: A randomized controlled study
Source: PLoS One. 2024 Nov 12;19(11):e0311792. doi: 10.1371/journal.pone.0311792 (PMC11556722; doi:10.1371/journal.pone.0311792)
Supplement: S1 File — (PDF) [file pone.0311792.s001.pdf]

S1: Intervention script

1. Thank you for joining me today! I am calling you for a 15/20 minutes chat to talk about anything you would like. With everything that is going on right now, our lab is concerned that some seniors might not get the attention that they should get during this really stressful and unprecedented time, and we thought it would be nice to reach out to seniors in the community and just have a conversation to keep you company and make you feel better.

2. Please let me know if at any time during this conversation you feel uncomfortable or you would prefer changing the topic of our talk.

3. To start, why don't you tell me more about yourself and I can tell you a little bit more about me as well....

• Demographics:

- Where were you born?
- Where do you live?
- Do you have any children/grandchildren?
- Do you have any pets?

• Past occupation

- What was your job?
- What was your first job?

• Hobbies

- Movies
- What are you currently watching on tv?
- What is your favorite movie/tv show?

    - Who is your favorite actor/actress?
- Food
    - Do you like to cook?
    - What recipe do could recommend trying?
    - What's your favorite type of restaurant?
    - Have you ever tried take-out food?
- Outside hobbies:
    - What do you do for fun outside?
    - Do you like fishing/gardening/walks?
    - Do you practice any sports?
    - Are you doing any exercises at home to stretch or move around?
- Reading:
    - Are you reading any books/magazines?
    - Do you like reading the newspaper?
- Feel good memories/background:
    - What's your favorite memory from your childhood?
    - What's your favorite memory from any traveling that you have done?
    - What was your first car?
- Arts:
    - Do you like to paint/draw?
    - Do you play any instruments? Did you in the past?
    - What music do you enjoy listening to?
    - Are you listening to any specific artists or albums during this quarantine that make you feel in a good mood?

- If you are listening to any of the current music, who is your favorite artist?
- Can you recommend any artists from the past that you think everyone should listen to today?

• Advice for us?

- What advice could you give our youth in the lab?
- How have you dealt with stress in your life?

What do you recommend doing to face challenges and maintain our wellbeing?
