# Supplementary material for: Impact of remote social interaction during the COVID-19 pandemic on the cognitive and psychological status of older adults with and without cognitive impairment: A randomized controlled study
Source: PLoS One. 2024 Nov 12;19(11):e0311792. doi: 10.1371/journal.pone.0311792 (PMC11556722; doi:10.1371/journal.pone.0311792)
Supplement: S3 File — (PDF) [file pone.0311792.s003.pdf]

<u>Research Protocol Narrative for “Impact of frequent social interaction through communication technologies in the cognitive status of socially-isolated older adults with and without cognitive impairment”</u> <u>(adapted from VHA Handbook 1202.1)</u>

(1) **Rationale**

(a) <u>Statement of the Problem.</u> AD is a neurodegenerative disorder that gradually destroys one’s ability to learn, reason, and carry out daily activities. Instead of thinking about it as a dementia, we now think about AD as a single pathologic entity with various stages, including preclinical, MCI, and dementia stages. The disease presents as dementia in 5% to 11% of people over the age of 65, affecting as many as 50% of those over the age of 85. The MCI stage is thought to be even more common, affecting up to 10-15% of those over the age of 65, and approximately 75% of those individuals will go onto develop Alzheimer’s disease dementia (Alzheimer’s Association, 2017). The Alzheimer's Association reported in 2017 that an estimated 5.5 million Americans are currently diagnosed with AD, and that the current direct and indirect costs for care are estimated at a staggering $259 billion annually. Given that efforts to develop new medications to alter the course of AD have not yet been successful, additional strategies are needed to improve the lives of the rapidly growing AD population in America, expected to reach 7.7 million in 2030 and between 11 and 16 million by 2050 (Alzheimer’s Association, 2017).

Several observational studies have associated different lifespan experiences such as physical activity, education, occupation, and social interaction with a protective effect in cognitive reserve later in life(1). Meaningful social connections have been associated with lower mortality, improved health outcomes and better quality of life(2–4). Previous studies have also suggested a negative impact of social isolation in cognitive outcomes in the elderly population (5–8). However, findings have been heterogeneous and limited by a use of different definitions and measures for social isolation and a variety of tested cognitive outcomes. Furthermore, there are no previous studies in humans that have assessed how fast and in what ways can social isolation affect cognition.

Communication technologies have been implemented in previous studies as a surrogate for social interaction to treat social isolation in older adults. However, results have been limited not only by the heterogeneity of the interventions but also by the difficulty in defining and controlling for social isolation in this population(9).

During the current pandemic, the policies to ensure public safety have included a widespread recommendation of staying home and avoiding any non-critical social contact. These policies have created a unique model of social isolation in which people are outside their usual social schedules and their interactions are limited to communication technologies, when available.

(b) Hypotheses or Key Question. The main aim of this study is to better understand the relationship between social interaction and cognitive function. We aim to establish the impact of frequent social interaction through communication technologies during COVID-19 pandemic in the cognitive status of socially-isolated older adults with and without cognitive impairment. We hypothesize that participants without frequent social interaction will perform worse on testing of executive functions, working memory, semantic memory, and delay recall, as compared to participants who have engaged regularly in social interaction through interactive communication technologies (ICT).

(c) Specific Objectives:There are two specific aims in this proposal. **Aim 1** will test the hypothesis that frequent social interaction through ICT during COVID-19 pandemic will have a significant positive impact in cognitive performance on testing. Experiment for Aim 1 will comprise a crossover design to compare an interventional phase of frequent social interactions through either telephone or online videoconferences between participants and researchers with a passive control phase where there will not be any interactions with the participants. Standarized cognitive testing will be compared at baseline and at the end of each phase. **Aim 2** will examine how social isolation and cognitive status influence misperceptions around the current pandemic. Experiment for Aim 2 will involve the administration of an adapted survey that evaluates knowledge and misperceptions regarding COVID 19(10). We will correlate the grade of misinformation with the severity of social isolation, and cognitive impairment.

(2) Background and Significance

(a) Background. Social isolation has been defined as the absence of social relationships and disengagement from the community(11). Research has shown that social isolation has a significant impact on frailty, healthcare outcomes and survival rates(8). Social isolation may be associated with poorer cognitive outcome and dementia later in life but results have been mixed(5,6,8). Several groups have found an association between living alone, feeling lonely or social disengagement with poorer cognitive outcomes on testing and higher risk of dementia later in life(6,12–15). A 50% increase in the risk of developing dementia at two years of follow up has been reported in some cohorts(16). However, other studies have found no association between living alone and cognitive decline(1,15). Previous studies have assessed different aspects of this phenomenon and have focused on either living alone, social isolation or feelings of loneliness. These three concepts, even though they may overlap, are distinct in nature, and might explain the difference in the results obtained. Longitudinal studies that have reported the association between social isolation, loneliness and cognitive decline have followed the participants' cognitive status for two, eight, and ten years(12,14,16). However, there are no previous studies in humans that have assessed how fast and in what ways can social isolation affect cognition.

The use of ICT to assess social isolation in the elderly has been evaluated in previous randomized controlled studies and observational cohort studies by using telephone befriending programs and web-based apps such as email, chat rooms, and videoconferences(9,17–19). Studies that have examined the impact of ICT on social isolation have looked at several aspects of it, such as loneliness, social connectivity, social well-being, and social support. The heterogeneity of the different definitions for the studies' outcomes limits the comparison of the results(9). Several

studies have shown a significant improvement in social isolation and some have associated ICT with a significant reduction of loneliness and depression(17,18). However, there is still a need for more well-designed studies to address the effect of ICT interventions on the treatment of social isolation in the elderly population.

(b) Significance. Depending on the definition, the prevalence of social isolation among people over 60 years of age has been described between 5 to 24%(9,15,20–22). Among veterans, loneliness has been reported with a prevalence of 10-44% and has been associated with greater age, disability and psychiatric disorders(23). AD in its MCI and mild dementia stages are major health issues affecting our Veterans. Because our Veteran population is aging more rapidly than that of the US as a whole, AD is of particular concern to Veterans and their families. In 2016 there were 9,846,441 Veterans over the age of 65, representing 45% of the Veteran population (Veterans Affairs, 2016). Given that in a population over the age of 65 AD dementia develops in 5% to 11% and MCI in up to 20%, there are currently between 492,322 and 1,083,105 Veterans with AD dementia, and up to 1,969,288 with MCI due to AD. The need to keep our Veterans with AD in the MCI and mild dementia stages as functional as possible is critically important. The proposed research first seeks to gain an increased understanding of the influence of social isolation and loneliness on cognition in healthy older adults and patients with aMCI and mild AD. Also, we will aim to evaluate the utility of ICT on cognitive performance, loneliness and psychiatric symptoms. The proposed intervention could potentially provide a drug-free, non-invasive method to help improve memory in patients with aMCI and AD while providing social support. Ultimately this behavioral intervention could be woven into VA programs such as Home-Based Primary Care and the Patient Aligned Care Teams.

c) Relevance to Veterans Health. Because our Veteran population is aging more rapidly than that of the US as a whole, AD is of particular concern to Veterans and their families. In 2016 there were 9,846,441 Veterans over the age of 65, representing 45% of the Veteran population (Veterans Affairs, 2016). Given that in a population over the age of 65 AD dementia develops in 5% to 11% and MCI in up to 20%, there are currently between 492,322 and 1,083,105 Veterans with AD dementia, and up to 1,969,288 with MCI due to AD. The need to keep our Veterans with AD in the MCI and mild dementia stages as functional as possible is critically important.

(3) Work Accomplished

Regarding the use of communication technology in the elderly population, there are several studies that have assessed the feasibility for this population to use and adhere to a behavioral intervention using this type of platforms. The PRISM study was a randomized controlled trial that evaluated the use of communication technology to improve social support in a group of 300 older adults living independently. After the intervention, they documented significantly less loneliness and increased perceived social support and well-being with only 10% of attrition(24). Other studies have reported a higher attrition rate of up to 40%(9). However, these participants were encouraged to use the technological interventions on their own without any assistance from

researchers. ICTs have been used in patients with MCI and mild dementia but its use has focused on assisting with safe walking, independent living, safe living and cognitive stimulation(25,26).

In our lab, we have carried out multiple behavioral experiments using technological interfaces in healthy older participants, patients with mild cognitive impairment, and mild dementia to assess different aspects of the cognitive abnormalities in Alzheimer's disease. We have ample experience administering and analyzing the proposed cognitive batteries for this study. Now, we aim to add a social intervention as an experiment to assess changes in cognitive performance in this population.

(4) **Work Proposed**

(a) In the current proposal, we are planning to perform an experimental crossover study to answer questions posed by our initial aim. The proposed research will take approximately 1 year, with the goal of enrolling approximately 150 total participants over the course of 1 year: 50 healthy older participants, 50 patients with amnestic mild cognitive impairment, and 50 patients with mild AD. We plan to recruit subjects from VA Boston who have previously participated in previous protocols in our lab and new participants from the memory clinic. For this, we will recruit previous participants from other studies from our lab who have signed a permission to re-contact. We will also recruit new patients from the Memory disorders clinic at Jamaica Plain and Brockton campuses. For this, we will request a HIPAA waiver for recruitment in order to review the patients' charts to determine eligibility.

(b & c) As previously mentioned above, there is one core experiment in this proposal. Experiments that fall under the themes and aims of this proposal may be added in the future (through future protocol amendments). In the following section, each specific aim, along with the associated experiment that fall within the purview of that aim, are outlined in more detail.

Aim 1: To assess the change from baseline to week 4 in a composite telephone cognitive battery when participants engage in regular social interaction through technological communication platforms during the intervention phase, as compared to no social interaction in the control phase.

The goal of Aim 1 is to test the following research question: Among older adults, with and without cognitive impairment, in social isolation due to COVID-19 pandemic, can regular social interaction through technological communication platforms improve their cognitive status?

To answer this question, we will perform an experimental study involving a crossover design. We will employ an AB/BA crossover design (2-sequence, 2-period, 2-treatment design) where A will be the intervention phase and B will be the passive control phase (Table 1). Each period will have a duration of 1 month. Assessment of our primary outcome will be done at the end of each period (Figure 1). Given that we will study a behavioral intervention, we consider that a 4-week washout period will suffice to observe an acute change in cognitive performance without significant carryover effects.

Table 1. 2x2 Crossover Design		
**Sequence**	**Period 1**	**Period 2**
AB	A	B
BA	B	A

Baseline and outcome evaluations
Figure 1 summarizes the assessments that will be given at baseline and after each intervention/control phase.Baseline and outcome evaluations will be done via telephone. For the baseline evaluation, we are planning to have two separate sessions to avoid participants' fatigue. First telephone session will take 1 hour. We will perform a telephonic interview to gather demographic characteristics including: age, living arrangement, marital status, level of education, ethnicity, current or former occupation, cognitive activities (e.g. reading, listening to the radio, playing chess or cards), functional status, mobility, hearing or visual impairments, social activities performed before and after current quarantine, access to communication technologies at home, and personal or familial impact by COVID19 (see baseline interview in appendix). We will also administer at this time the telephone Montreal Cognitive Asessment (MoCA), and validated scales to assess social isolation (Lubben social isolation scale), depression (Geriatric depression scale) and anxiety (Geriatric Anxiety Inventory).
Second telephone session for baseline evaluation will be done at participants' convenience but ideally will occur the next day. We anticipate that this second session will take 40 minutes to complete. At this time, we will complete a phone-administered neuropsychological battery (see details on the next section), a validated scale to assess feelings of loneliness (UCLA loneliness scale), and a Risk perception scale and a misinformation survey regarding current COVID-19 pandemic. The risk perception scale and misinformation surveys were adapted from a recently published study assessing risk perception in current COVID19 pandemic(27) (see appendix) and a COVID19 survey to address misinformation around current pandemic (explained in Aim 2).

Primary outcome assessment: Cognitive evaluation
Cognition will be measured through a phone-administered neuropsychological battery which will be administered at baseline and after the end of the control and intervention phase (at 1 month and at 2 months). To avoid practice effect from repetitive evaluations, we will use several subtests from the Repeatable battery for the Assessment of Neuropsychological Status (RBANS)(28): List learning, recall, recognition, semantic fluency, and digit span. We will use a different form for each assessment which contains different word lists, categories, and digit sequences. For the phonemic fluency to letters, we will use a different pair of letters for each assessment. We will also administer the Weschler Memory scale-III (WMS-III) orientation subtest and the oral trail making test. Validity: The RBANS has been previously validated for remote administration in 18 adult subjects over age 55 with and without cognitive impairment with correlations that ranged from 0.75 to 0.90 for the subtests that will be used for this study(29). OTMT-B has been previously validated as an alternative form for written TMT-B and has been used during fMRI studies with strong activation of dorsolateral and medial prefrontal cortices(30–32). No studies have been reported assessing its remote use.
The proposed battery will include (see appendix):

- Orientation (WMS-III)

- RBANs subtests
    - List learning
    - List recall
    - List recognition
    - Digit span – attention and working memory
    - Categories- semantic processing (activation retrieval)
- Letters – phonemic verbal fluency
- Oral trail making test A and B (OTMT)

Secondary outcomes assessment:
We plan to assess, as a secondary outcome, the change from baseline to week 4 in psychiatric symptoms using the Geriatric depression scale, Geriatric Anxiety Inventory and the UCLA loneliness scale when participants engage in regular social interaction through technological communication platforms during the intervention phase as compared to no social interaction in the control phase. For this analysis, these scales will be administered at baseline and after the end of the control and intervention phase (at 1 month and at 2 months). We will also include the risk perception scale, and Lubben social isolation scale to assess current worry around the COVID19 pandemic and level of social isolation throughout the study, which might influence their cognitive performance. In this study, we defined social isolation as a state in which an individual has a minimal number of social contacts and lacks engagement with others either physically or remotely through communication technology. To assess social isolation, we will use the 6-item version of the Lubben Social Network scale, which is a widely accepted validated scale for measuring social isolation. We defined social isolation as a total score below 12, as suggested by the authors(11).

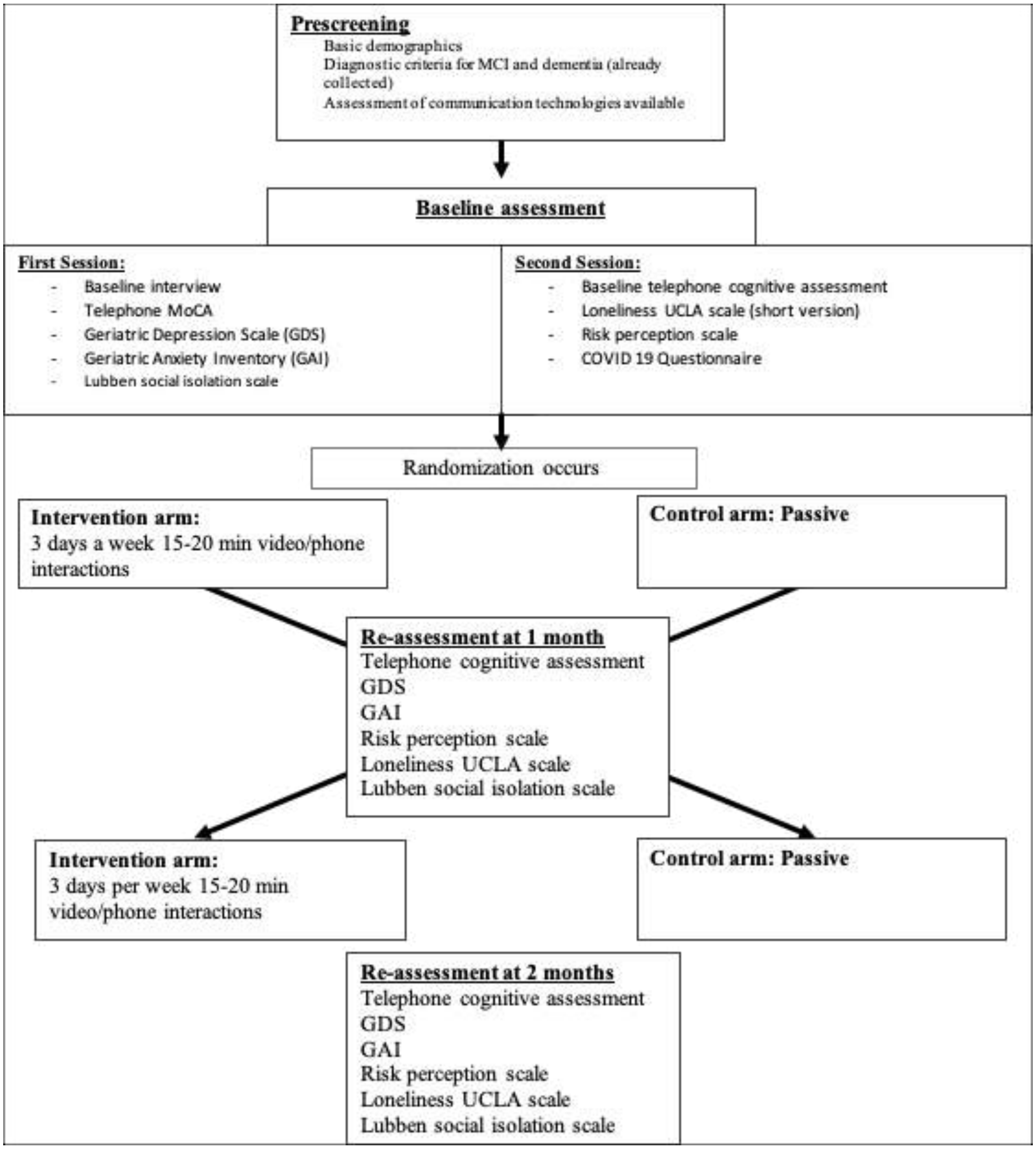

Figure 1: Baseline and outcomes assessment

Randomization and Counterbalancing

To avoid imbalance among groups, randomization will be stratified by cognitive syndrome diagnosis (healthy older adult, mild cognitive impairment and mild dementia). Participants will be randomized to the treatment sequences using a 1:1 allocation ratio **(Table 2**). Outcome assessors will be blinded to the treatment randomization and allocation. Randomization and allocation of the treatment sequence will be done by a project coordinator who will not be part of administering the intervention or the baseline and outcomes assessments.

**Table 2. Crossover Design stratified by diagnosis**						
**Sequence**	**Period 1 (First month)**			**Period 2 (second month)**		
	Healthy Controls	MCI	Dementia	Healthy Controls	MCI	Dementia
AB	A	A	A	B	B	B
BA	B	B	B	A	A	A

We will also do a counterbalancing of the three versions of the RBANs that will be used among the three categories. We will have eight assessors for the study who will be assigned a participant in a sequential order by the project coordinator. Project manager will also assign the order of version to be used by the assessor for each participant. The project manager will use the following sequence for the different versions of the RBANs:

- First participant will get version A at baseline, version B at first month and version C at second month
- Second participant will get version B at baseline, version C at first month and version A at second month
- Third participant will get version C at baseline, version A at first month and version B at second month
- Fourth participant will start again the sequence A-B-C and so on.

This counterbalancing will be done for each diagnosis separately (**Table 3**).

**Table 3. Counterbalancing the different versions of the RBANs by diagnosis**									
	**Sequence of RBANs versions to be used**								
	**Healthy controls**			**MCI**			**Dementia**		
**Baseline**	A	B	C	A	B	C	A	B	C
**First month**	B	C	A	B	C	A	B	C	A
**Second month**	C	A	B	C	A	B	C	A	B

Intervention phase

The proposed intervention will work to assess the acute cognitive impact associated with social isolation by providing more social interaction between individuals during a time of social distancing and highly limited in-person social interactions. Participants will engage in social conversations with members of the research study staff over scheduled videoconferencing calls or through telephone calls three times per week. The use of either videoconference or telephone, will depend on the availability of the technologies at the participant's home and his/her preference. If the participants are able to participate in a videoconference call, we will use the following web-based apps: Cisco Webex, Microsoft teams or Skype. Research staff will guide the conversation using a script designed to both promote pleasant interaction as well as gather valuable demographic information in which we are interested (see intervention talking points in Appendix). Social interactions will be designed to be approximately 20 minutes in length and covering participants' remote history, current issues/events, personal interim events/history, media sources, participants' hobbies/interests, and other spontaneous topics of interest.

Control phase:
Given that we want to assess the impact of current social isolation model during current COVID19 pandemic, we will compare our intervention phase with a passive control phase. During the control phase, the participants will not have any contact with the research team over the phone or video platforms. They will be instructed to go back to their usual routine which currently is restricted to a stay-at-home and social-distancing advisory from the state.

Analyses:
Data will be analyzed with Stata 16. Differences from baseline in R-BANS subtests, Oral TMT-B, and phonemic fluency will be compared between the intervention phase and control phase in all subjects with an unpaired t-test, as these cognitive tests are predicted to present a normal distribution. We will correct for multiple comparisons using Bonferroni correction. For the secondary outcomes, changes in GDS, GAI, and loneliness scale will be compared among the intervention and control phase using an unpaired t-test.

Aim 2: will assess the correlation between social isolation, severity of cognitive impairment assessed by a telephone-administered cognitive battery and its correlation with the level of misinformation around COVID19 pandemic in healthy older adults, MCI and dementia due to AD. Our hypothesis is that there will be an inverse correlation between the level of misinformation around current pandemic and the Lubben social isolation scale and composite cognitive scores. To test this hypothesis, during the telephone baseline assessment, we will administer a questionnaire that evaluates knowledge and misperceptions regarding COVID 19. This questionnaire has been adapted from a previously published survey(10). We will correlate the grade of misinformation with the severity of social isolation, and cognitive performance at baseline.
Analyses: We will correlate the level of misinformation around COVID 19, social isolation, and cognitive impairment by using Pearson correlation and logistic regression.

(d) There is one main potential limitation to the proposed research. The methods used in the proposed research may cause patients to become fatigued or frustrated. Regular social interaction with research staff might become monotonous and participants might become disengaged from the experiment. Research staff will make full efforts to make participants as comfortable as possible (see Human Studies Section for additional details).

(e) **Power Analyses.** Sample size calculation was performed using Stata 16 (Stata Corp LLC, Texas, USA). We are expecting a small to medium effect from the social intervention. By calculating an effect size of 0.35, with a SD of 2.3 on RBANS subtests for attention and memory, and a significance level of 0.05 and power of 0.8, our calculated sample size is of 131 participants. Expecting a 10% of attrition based on previous literature(24), we will recruit a total of 150 participants.

k. **Human Studies Section**

(1) **Risk to Subjects**

Human Subjects Involvement and Characteristics. We plan to enroll an estimated 150 participants. Patients will come from VA Boston. Participants from prior studies from our lab, who have previously signed a permission to contact, will be contacted by letter to recruit. With a HIPAA waiver, study staff authorized by the IRB will access CPRS to identify potential participants from the Memory clinic at JP and Brockton campuses. Then if subjects are eligible, we will send a recruitment letter to invite them to the study.. . Healthy older adults, patients with MCI, and patients with AD will complete standardized testing to determine a research grade diagnosis for appropriate group categorization. For participants from prior studies, with a HIPAA waiver, we will review prior cognitive assessments to determine an appropriate categorization. For both new participants and prior participants, we will complete a telephone MoCA during baseline assessment to determine status of participants prior randomization.

Inclusion Criteria

1. Participants will be assigned to one of three groups based on the inclusion criteria summarized below.

a) **Healthy older adults:** Will be 50-100 years of age; Performance within 1.0 SD for age & education adjusted norms on our internal neuropsychological test battery (using norms derived from Weintraub et al., 2009); MoCA > 26 (Nasreddine et al., 2005). Norms for our neuropsychological test battery are based off of each individual test, and previously established norms used by others (Weintraub et al., 2009). Healthy older controls must have no history of depression, alcohol or substance abuse, cerebrovascular disease, traumatic brain damage, stroke, or any other type of focal brain damage, or any other type of neurodegenerative disease (e.g., Parkinson's). These latter conditions will only be obtained by self-report.

b) **Mild AD dementia**: Meets probable AD dementia NIA-AA criteria(33); 50-100 years of age; MoCA 15-25; performance on delayed recall and recognition memory worse than 1.5 SD for age and education; worse than 1.5 SD for age & education in at least one other cognitive domain (e.g., language, executive functioning) based on other tests in our neuropsychological test battery. Dr. Budson will confirm all mild AD dementia diagnoses.

c) **MCI due to AD**: Meets MCI due to AD NIA-AA criteria(34); 50-100 years of age; MoCA >18; performance on delayed recall and recognition memory worse than 1.0 SD for age & education adjusted norms. Dr. Budson will confirm all MCI due to AD diagnoses.

2. Subjects will need to have access to either a computer, smart device, or telephone to be able to participate in the social interaction section of the study by video or audio phone call.

.

(a) Exclusion Criteria: Subjects with a clinically significant problem of any of the following conditions will be excluded: depression, alcohol or drug use, cerebrovascular disease, traumatic brain damage, or any non-neurological condition whose severity could significantly impair cognition (e.g., organ failure). Subjects will be excluded if they cannot understand the informed consent. Subjects will be excluded if they have a significant hearing impairment which will prevent them from understanding the informed consent and from completing the experiment.

Inclusion of Women and Minorities

All subjects meeting the inclusion/exclusion criteria will be entered in the study, regardless of race, gender, and ethnic or socioeconomic background, to allow the results of this research to yield the greatest generalizability.

Inclusion of Children

Children do not develop Alzheimer's disease; therefore, they will not be included in this study.

(b) Sources of Materials. Information solicited from *patients* will include age, gender, level of education, medical history, medical records, data from neuropsychological testing, and questionnaires. Information solicited from *healthy controls* will be the same, except we will not use information from their medical records. If the participant is a VA patient, clinical staff authorized by IRB and with CPRS access will access their medical record to confirm a clinical diagnosis. For healthy controls we will only use participants that had participated in previous studies at our labs, were categorized in that group, and had signed a permission to contact form.. With a HIPAA waiver, we will review prior collected history and prior cognitive assessments from other studies to determine an appropriate categorization. All data and demographics collected in this study will only be used for the purposes of this research. All data obtained as part of this research project will be coded, will not contain personally identifying information regarding the participant and will be stored securely in a locked file cabinet in the laboratory and/or behind a firewall on password-protected computer.

(c) Potential Risks. Care is taken in the process of recruitment and during the conduct of research to protect participants' well-being and privacy. No deception is used in this research. All research staff involved with this study will be thoroughly trained to answer any questions, should they arise, and every effort will be made to make this process as comfortable as possible for participants. Strict measures will be taken to maximize participant privacy. All data obtained as part of this research project including neuropsychological testing, and questionnaires will be coded, will not contain personally identifying information, and will be stored securely in a locked file cabinet. Identifiable data gathered to determine inclusion or exclusion and participant categorization will be stored in a separate locked filing cabinet located in a separate area of the laboratory. Digital data will be saved electronically behind a firewall on a password-protected computers and server.

(2) Adequacy of Protection from Risks

Recruitment and Informed Consent. AD and MCI participants will be recruited from VA Boston. Participants from prior studies from our lab, who have previously signed a permission to contact, will be contacted by letter to recruit. With a HIPAA waiver, study staff authorized by the IRB will access CPRS to identify potential participants from the Memory clinic at JP and Brockton campuses. Then if subjects are eligible, we will send a recruitment letter to invite them to the study. All patients are assured that opting not to participate involves no loss or penalty to their health benefits, and if they do choose to participate, they can discontinue their participation at any time, for any reason.

Below is the outline of our procedures and pre-consent talking points when a patient in the clinic is identified for the first time and approached for research in our laboratory.

For patient recruitment:

1. Dr. Budson and/or other IRB approved Memory Disorders Clinic staff identify a patient that is potentially good for our research studies and falls within the inclusion criteria for the research protocol.

2. If the patient falls within inclusion criteria, Dr. Budson and/or other IRB approved Memory Disorders Clinic staff will alert the research staff in Dr. Budson's laboratory.

3. The research staff will verify the subjects eligibility via CPRS under a HIPAA waiver and will send a recruitment letter to the potential participants with the lab's contact information.
4. If the patient is interested in the research and calls the lab, the research staff will provide more information about research participation in Dr. Budson's laboratory and, importantly, will provide an additional opportunity for participants to decline participation. Information provided will consist of the following points:
    a. The research in Dr. Budson's laboratory focuses on ways to understand and improve thinking and memory in normal aging and in those with certain brain disorders.
    b. Individuals are first tested over the phone with tasks of memory to determine their general thinking and memory abilities.
    c. Individuals are administered several questionnaires to document the participants social support, functionality and daily activities.
    d. Then individuals are asked to interact with the researchers via telephone or video-conference using computer-based apps such as Cisco Webex, Microsoft teams and Skype.
    e. These regular interactions will take place three times per week, each lasting about 20 minutes.
    f. Sessions will be conducted within the comfort of their own home.

5. Potential participants are then given as much time as needed to decide whether or not they would be interested in participating. If they do not have an immediate answer, they are given the contact information for the post-doctoral fellow or laboratory manager. They can contact the post-doctoral fellow or laboratory manager if they decide they are interested in participating in research.

6. If the potential participant is interested in participating, he or she will either participate in the informed consent process using the initial consent form at this point, or will schedule an appropriate time to participate in the informed consent process.

For the recruitment of healthy older controls:

For healthy controls we will only use participants that had participated in previous studies at our lab, were categorized in that group at that time, and signed a permission to contact form. With a HIPAA waiver, we will review prior collected history and prior cognitive assessments from other studies to determine an appropriate categorization. We will send a recruitment letter to potential participants.

1. If a healthy older control calls the lab and indicates interest in participating, research staff will then provide them information regarding the study. Information will consist of the following points:
   a. The research in Dr. Budson's laboratory focuses on ways to understand and improve thinking and memory in normal aging and in those with certain brain disorders.
   b. Individuals are first tested over the phone with tasks of memory to determine their general thinking and memory abilities.
   c. Individuals are administered several questionnaires to document the participants social support, functionality and daily activities.
   d. Then individuals are asked to interact with the researchers via telephone or video-conference using computer-based apps such as Cisco Webex, Microsoft teams and Skype.
   e. These regular interactions will take place three times per week, each lasting about 20 minutes.
   f. Sessions will be conducted within the comfort of their own home

2. Potential healthy older control participants are then given as much time as needed to decide whether or not they would be interested in participating. If they do not have an immediate answer, they are given the contact information for the post-doctoral fellow or laboratory manager. They can contact the post-doctoral fellow or laboratory manager if they decide they are interested in participating in research.

Given current social restrictions due to COVID-19, the informed consent process will be done over the telephone. During the informed consent process, the research protocol will be explained in simple but accurate language. For those with cognitive impairment, the study is broken down into different sections and approved staff members will verify if the participant understands and is interested in participating. It is emphasized that any questions they have will be answered before they begin, and that they are free to withdraw from the study at any time, for any reason. Given the current COVID-19 pandemic and the social distancing procedures that have been put in place we will waive documentation of consent in order to allow participants to agree to participate without having to leave their household during the current circumstances. The consent form procedure will not ask participants to sign the consent form, but verbal consent will be required. Without this alteration to the consent form it will not be practicably feasible to ask our participants to mail the hard copies of signed consent forms back to us. A copy of the consent documents will be mailed to the participants, or will be electronically emailed to them. Once we obtain the verbal consent, we will proceed to enroll the participant.

a) Protection Against Risk. To protect participant confidentiality the data files are assigned a unique number and the names and other HIPAA identifiers are not used. Subjects are identified by name only on the consent and HIPAA form, which are kept separate from the rest of the data obtained as part of this research project in a locked file cabinet. All subject names, contact information, and the title of the experiment they participated in are kept in an entirely separate, password-protected, electronic database on a VA server behind the firewall. A master linking file that identifies a coded information to a specific subject will also be stored in a separate, password-protected, electronic database on a VA server, behind the firewall. The master linking file will also be password protected itself, and will only be accessible to IRB-approved research staff members. The purpose of this database is to avoid rescheduling subjects for the same study or a different study that uses the same materials. Participants will be informed at the beginning of the study that they are free to terminate their participation at any time, and that this discontinuation will in no way influence their treatment or relationship with health care providers at any institution. Highly trained study staff members will be working with subjects throughout the study and will take the appropriate steps to minimize anxiety and fatigue.

(3) Potential Benefit of the Proposed Research to the Subject and Others.

There are no direct benefits to the subjects from participating in the research. However, the proposed research may lead to new methods or interventions that will potentially enable patients improve their memory, social support and mood. Behavioral experiments pose minimal risk to patients. Patients may experience fatigue or frustration, and trained study staff will the appropriate steps to minimize this.

(4) Importance of the Knowledge to be Gained

In addition to theoretical importance, the proposed research may provide new methods to decrease social isolation and improve cognitive performance that may help improve the quality of life for patients and their caregivers.

(5) **<u>Resources</u>**

a) <u>Research Space:</u> Dr. Budson's laboratory is at the VA Boston Healthcare System, Jamaica Plain campus, 12th floor. The laboratory consists of a large room for research assistants and students with a single workstation. There are four offices for postdoctoral fellows and other trainees. Each office is of ample size, and are furnished with two desks, file cabinets, and white boards. There is a large testing room with two testing chambers. One chamber is used for general behavioral testing, and the other is equipped with a 128-channel Biosemi Active Two data acquisition system used for electroencephalography (EEG) and event related potential experiments (ERP). The Active Two system uses active electrodes, and is capable of 24-bit continuous sampling at 4,096 samples per second. Active electrodes are amplified at the source, eliminating the need for abrading of the skin and measuring skin-electrode resistance. ActiView software is used for data acquisition. Post-processing is performed using the EMSE Suite (Source Signal Imaging, San Diego, CA). This set up is connected to two Windows-based computers, one used for data acquisition and the other used for data presentation. In addition to these EEG and ERP computers, Dr. Budson's laboratory consists of 2 Dell Latitude E6510 laptops and 2 Dell Inspiron laptops. Each laptop is equipped with E-Prime (used to conducted behavioral experiments), statistical software (SPSS), Adobe Acrobat, Microsoft Office Suite, and the requisite software for EEG / ERP experiments. Softwares to be used in this study are already in place for all lab studies, and no additional licences will be required.

However, due to current COVID-19 pandemic, most of the staff from the lab is working remotely from their homes. The advanced fellow in Neurology, who is part of the IRB approved research staff, is still coming to the lab on a regular schedule and she will be in charge of keeping the records for the participants' consent forms and all other documents that contain personally identifying information in the assigned locked cabinet in the lab.

Web Applications

For the intervention phase, we will use the following web applications: Cisco Webex, Microsoft teams and Skype. The choice of web application used will depend on the preference of the participant.

Data on Hard Drive

Data will not be stored on PC Hard Drive.

Mobile Devices

All mobile/portable devices and media will be protected with VA Approved FIPS 140-2 compliant technology.

Storage Location

The PI will work with VABHS IRM, once the study is approved, to create an appropriate folder for electronic study files on a server approved to hold research data. Once the folder is being created, the PI will report the network location/server name to ISOs and Research Administration.

Removal of VA Sensitive Information from the VA Protected Environment
Data will not be removed from the VA protected environment at any time.

Data Transmission
Data will be securely transmitted using the VA approved methods. We will use FIPS 140-2 validating encryption.

Data Backup
Mobile storage devices will not the only copy of research information. Origianal electronic VA data will be backed up regularly and stored behind the VA Firewall.
Termination of Data Access
Once study team members are no longer a part of the research team, their access to data and research materials will be terminated.

Incident Reporting
Suspected Information security and privacy incidents will be reported within one hour to the Information Security and Privacy Officers and Research Administration.

(b) **Other Research Resources:** N/A

n. **<u>Publications from Last Funding Period (as applicable).</u>** N/A

o. **<u>Literature Citations (as applicable).</u>**

1. Evans IEM, Llewellyn DJ, Matthews FE, Woods RT, Brayne C, Clare L, et al. Social isolation, cognitive reserve, and cognition in healthy older people. PloS One. 2018;13(8):e0201008.

2. Rowe JW, Kahn RL. Successful aging. The Gerontologist. 1997 Aug;37(4):433–40.

3. Holt-Lunstad J, Smith TB, Layton JB. Social relationships and mortality risk: a meta-analytic review. PLoS Med. 2010 Jul 27;7(7):e1000316.

4. Cornwell EY, Waite LJ. Social disconnectedness, perceived isolation, and health among older adults. J Health Soc Behav. 2009 Mar;50(1):31–48.

5. DiNapoli EA, Wu B, Scogin F. Social isolation and cognitive function in Appalachian older adults. Res Aging. 2014 Mar;36(2):161–79.

6. Holwerda TJ, Deeg DJH, Beekman ATF, van Tilburg TG, Stek ML, Jonker C, et al. Feelings of loneliness, but not social isolation, predict dementia onset: results from the Amsterdam Study of the Elderly (AMSTEL). J Neurol Neurosurg Psychiatry. 2014 Feb;85(2):135–42.

7. Shankar A, Hamer M, McMunn A, Steptoe A. Social isolation and loneliness: relationships with cognitive function during 4 years of follow-up in the English Longitudinal Study of Ageing. Psychosom Med. 2013 Feb;75(2):161–70.

8. Evans IEM, Martyr A, Collins R, Brayne C, Clare L. Social Isolation and Cognitive Function in Later Life: A Systematic Review and Meta-Analysis. J Alzheimers Dis JAD. 2019;70(s1):S119–44.

9. Chen Y-RR, Schulz PJ. The Effect of Information Communication Technology Interventions on Reducing Social Isolation in the Elderly: A Systematic Review. J Med Internet Res. 2016 Jan 28;18(1):e18.

10. Geldsetzer P. Knowledge and Perceptions of COVID-19 Among the General Public in the United States and the United Kingdom: A Cross-sectional Online Survey. Ann Intern Med. 2020 Mar 20;

11. Lubben J, Blozik E, Gillmann G, Iliffe S, von Renteln Kruse W, Beck JC, et al. Performance of an abbreviated version of the Lubben Social Network Scale among three European community-dwelling older adult populations. The Gerontologist. 2006 Aug;46(4):503–13.

12. van Gelder BM, Tijhuis M, Kalmijn S, Giampaoli S, Nissinen A, Kromhout D. Marital Status and Living Situation During a 5-Year Period Are Associated With a Subsequent 10-Year Cognitive Decline in Older Men: The FINE Study. J Gerontol Ser B. 2006 Jul 1;61(4):P213–9.

13. Yaffe K, Fiocco AJ, Lindquist K, Vittinghoff E, Simonsick EM, Newman AB, et al. Predictors of maintaining cognitive function in older adults: the Health ABC study. Neurology. 2009 Jun 9;72(23):2029–35.

14. Mazzuco S, Meggiolaro S, Ongaro F, Toffolutti V. Living arrangement and cognitive decline among older people in Europe. Ageing Soc. 2017 Jul;37(6):1111–33.

15. Evans IEM, Llewellyn DJ, Matthews FE, Woods RT, Brayne C, Clare L, et al. Living alone and cognitive function in later life. Arch Gerontol Geriatr. 2019 Apr;81:222–33.

16. Grande G, Vetrano DL, Cova I, Pomati S, Mattavelli D, Maggiore L, et al. Living Alone and Dementia Incidence: A Clinical-Based Study in People With Mild Cognitive Impairment. J Geriatr Psychiatry Neurol. 2018;31(3):107–13.

17. Cattan M, Kime N, Bagnall A-M. The use of telephone befriending in low level support for socially isolated older people--an evaluation. Health Soc Care Community. 2011 Mar;19(2):198–206.

18. Cotten SR, Anderson WA, McCullough BM. Impact of internet use on loneliness and contact with others among older adults: cross-sectional analysis. J Med Internet Res. 2013 Feb 28;15(2):e39.

19. Neil-Sztramko SE, Coletta G, Dobbins M, Marr S. Impact of the AGE-ON Tablet Training Program on Social Isolation, Loneliness, and Attitudes Toward Technology in Older Adults: Single-Group Pre-Post Study. JMIR Aging. 2020 Apr 20;3(1):e18398.

20. Gale CR, Westbury L, Cooper C. Social isolation and loneliness as risk factors for the progression of frailty: the English Longitudinal Study of Ageing. Age Ageing. 2018 May 1;47(3):392–7.

21. Kuiper JS, Zuidersma M, Zuidema SU, Burgerhof JG, Stolk RP, Oude Voshaar RC, et al. Social relationships and cognitive decline: a systematic review and meta-analysis of longitudinal cohort studies. Int J Epidemiol. 2016;45(4):1169–206.

22. Taylor HO, Taylor RJ, Nguyen AW, Chatters L. Social Isolation, Depression, and Psychological Distress Among Older Adults. J Aging Health. 2018;30(2):229–46.

23. Kuwert P, Knaevelsrud C, Pietrzak RH. Loneliness among older veterans in the United States: results from the National Health and Resilience in Veterans Study. Am J Geriatr Psychiatry Off J Am Assoc Geriatr Psychiatry. 2014 Jun;22(6):564–9.

24. Czaja SJ, Boot WR, Charness N, Rogers WA, Sharit J. Improving Social Support for Older Adults Through Technology: Findings From the PRISM Randomized Controlled Trial. The Gerontologist. 2018 08;58(3):467–77.

25. Holthe T, Halvorsrud L, Karterud D, Hoel K-A, Lund A. Usability and acceptability of technology for community-dwelling older adults with mild cognitive impairment and dementia: a systematic literature review. Clin Interv Aging. 2018 May 4;13:863–86.

26. D'Onofrio G, Sancarlo D, Ricciardi F, Panza F, Seripa D, Cavallo F, et al. Information and Communication Technologies for the Activities of Daily Living in Older Patients with Dementia: A Systematic Review. J Alzheimers Dis JAD. 2017;57(3):927–35.

27. Gerhold L. COVID-19: Risk perception and Coping strategies. 2020 Mar 25 [cited 2020 Apr 29]; Available from: https://psyarxiv.com/xmpk4/

28. Randolph C, Tierney MC, Mohr E, Chase TN. The Repeatable Battery for the Assessment of Neuropsychological Status (RBANS): preliminary clinical validity. J Clin Exp Neuropsychol. 1998 Jun;20(3):310–9.

29. Galusha-Glasscock JM, Horton DK, Weiner MF, Cullum CM. Video Teleconference Administration of the Repeatable Battery for the Assessment of Neuropsychological Status. Arch Clin Neuropsychol Off J Natl Acad Neuropsychol. 2016 Feb;31(1):8–11.

30. Mrazik M, Millis S, Drane DL. The oral trail making test: effects of age and concurrent validity. Arch Clin Neuropsychol Off J Natl Acad Neuropsychol. 2010 May;25(3):236–43.

31. Rane S, Caroselli JS, Dickinson M, Tran K, Kuang F, Hiscock M. Initial Development of a Modified Trail Making Test for Individuals with Impaired Manual Functioning. Appl Neuropsychol Adult. 2016;23(2):141–50.

32. Moll J, de Oliveira-Souza R, Moll FT, Bramati IE, Andreiuolo PA. The cerebral correlates of set-shifting: an fMRI study of the trail making test. Arq Neuropsiquiatr. 2002 Dec;60(4):900–5.

33. McKhann GM, Knopman DS, Chertkow H, Hyman BT, Jack CR, Kawas CH, et al. The diagnosis of dementia due to Alzheimer's disease: recommendations from the National Institute on Aging-Alzheimer's Association workgroups on diagnostic guidelines for Alzheimer's disease. Alzheimers Dement J Alzheimers Assoc. 2011 May;7(3):263–9.

34. Albert MS, DeKosky ST, Dickson D, Dubois B, Feldman HH, Fox NC, et al. The diagnosis of mild cognitive impairment due to Alzheimer's disease: recommendations from the National Institute on Aging-Alzheimer's Association workgroups on diagnostic guidelines for Alzheimer's disease. Alzheimers Dement J Alzheimers Assoc. 2011 May;7(3):270–9.
